# Luteolin Prevents UVB-Induced Skin Photoaging Damage by Modulating SIRT3/ROS/MAPK Signaling: An *in vitro* and *in vivo* Studies

**DOI:** 10.3389/fphar.2021.728261

**Published:** 2021-08-30

**Authors:** Jing Mu, Huisheng Ma, Hong Chen, Xiaoxia Zhang, Mengyi Ye

**Affiliations:** School of Traditional Chinese Medicine, Ningxia Medical University, Yinchuan, China

**Keywords:** UVB photoaging, MAPK, MMPs, luteolin, ROS

## Abstract

The aim of this study was to investigate the role of luteolin in the mechanism of ultraviolet radiation B (UVB)-induced photoaging. An *in vivo* photoaging model was established using UVB irradiation of bare skin on the back of rats, and an *in vitro* photoaging model was established using UVB irradiation of human dermal fibroblasts (HDF). Skin damage was observed using hematoxylin-eosin (HE) and Masson staining, skin and cellular reactive oxygen species (ROS) levels were detected by DHE and DCF fluorescent probes, mitochondrial membrane potential was detected by JC-1 staining, and protein expressions were detected by immunofluorescence and Western Blot. Results from animal experiments showed that luteolin reduced UVB-induced erythema and wrinkle formation. Results from cellular assays showed that luteolin inhibited UVB-induced decrease in cell viability. In addition, *in vitro* and *in vivo* experiments showed that luteolin reduced oxidative stress levels, decreased activation of matrix metalloproteinases (MMPs) and increased collagen expression. Continued cellular experiments using 3-TYP, an inhibitor of Sirtuin 3 (SIRT3), revealed a loss of cellular protection by luteolin and a decrease in collagen, suggesting that luteolin acts by targeting and promoting SIRT3. luteolin is involved in the protection of skin cells against UVB radiation-induced ageing via the SIRT3/ROS/mitogen-activated protein kinases (MAPK) axis and it may be a promising therapeutic agent for the prevention of UVB photoaging.

## Introduction

The skin is the primary organ that protects and prevents the body from environmental damage (Georgetti et al., 2013). Due to the reduction of the stratospheric ozone layer, the level of ultraviolet (UV) radiation reaching the Earth’s surface has increased. The effects of UV radiation on the skin have received increasing attention as it is the major environmental trigger affecting the structure of the skin (Lee et al., 2018). UV radiation comes from the electromagnetic spectrum of the sun and it consists of three main regions, UVA (315–400 nm), UVB (280–315 nm) and UVC (100–280 nm) (Slominski and Pawelek, 1998). UVC is almost completely removed by the ozone layer once it reaches the Earth; although both UVB and UVA reach the earth, UVB has a higher energy level than UVA and has a greater impact on the skin (Kwon et al., 2019).

Specifically, UVB radiation is a major cause of inflammation, oxidative stress and DNA damage, and it can cause damage to the skin’s elastic and collage n fibers, leading to the appearance of wrinkles (Egbert et al., 2014; Che et al., 2017). UVB radiation also releases hydroxyl radicals, which causes changes in cellular redox homeostasis and lead to the formation of lipid radicals, which in turn cause oxidative stress (Ogura et al., 1991). Serval studies have reported that inhibition of oxidative stress can reduce the damage caused by UVB exposure *in vitro* and *in vivo* (Cho et al., 2017; Duan et al., 2019). For this reason, natural antioxidants from plants have attracted great interest.

Luteolin is a natural flavonoid found in many plants such as carrots, broccoli and celery (Chen et al., 2012; Lim et al., 2013), with anti-inflammatory (Taliou et al., 2013), antioxidant (Manju et al., 2005) and anti-tumor (Lin et al., 2008) physiological activities. Studies have shown that flavonoids have physiological activity in skin protection against UVB radiation-induced erythema and cytotoxicity (Bonina et al., 1996). The physiological activity of luteolin has been extensively studied to protect fibroblasts from UVB damage by inhibiting inflammatory factors (IL-6, IL-1, TNF-α) and modulating several signaling pathways including NF-κB, AP-1 and JAK-STAT (Gendrisch et al., 2021). Therefore, we hypothesized that luteolin has a protective effect against UVB-induced skin barrier damage in SD rats and Human dermal fibroblasts cells. In the present study, we confirmed the protective effect of luteolin against UVB-induced photodamage in both *in vivo* and *ex vivo* models by expressions of related proteins, serum levels of inflammatory factors and examination of pathological sections of rat skin, and explored the possible pathways involved. These results provide a theoretical basis for luteolin to protect the skin from the effects caused by UVB irradiation.

## Materials and Methods

### Reagents and Antibodies

Luteolin (purity ≥98%, L812409) was purchased from Macklin Biochemical Technology Co., Ltd. (Shanghai, China). Malondialdehyde (MDA, A003-1-2) assay kit, superoxide dismutase (SOD, A001-3-2, EC No.1.15.1.1) assay kit and lactate dehydrogenase (LDH, A020-2-2, EC No.1.1.1.27) assay kit were all purchased from Nanjing Jiancheng Bioengineering Institute. SA-β-Gal assay kit (BC2580) was purchased from Beijing Solarbio Technology Co., Ltd. Fetal bovine serum (FBS) was purchased from Gibco (USA). Anti-SIRT3 (ab246522), anti-p-P38 MAPK (ab126425), anti-p-JNK (ab124956), anti-c-Jun (ab40766), anti-MMP-1 (ab134184), anti-MMP-3 (ab52915), anti-TGF-β (ab215715), anti-Smad3 (ab40854) antibodies were obtained from Abcam (China). Anti-Collagen I (AF7001) was purchased from Affinity Biosciences (USA). Secondary antibody for goat anti-rabbit immunoglobulin (IgG) horseradish peroxidase (HRP) were purchased from Abcam.

### Animals and Experimental Design

Healthy adult Sprague-Dawley (SD) rats (180–220 g) were purchased from Jiangsu ALF Biotechnology Co., LTD. The rats were housed under standard conditions (12 h light/12 h dark cycle) and fed and watered ad libitum. All the procedures and experimental protocols were approved by the Ethics Committee for Animal Experiments of Ningxia Medical University, and the protocol number of authorization for the use of animals was No. 2021-008.

After 1 week of acclimatization, the rats were randomly divided into five groups (*n* = 8 per group): Group 1 (Ctr): Rats were kept under normal conditions. Group 2 (Mod): Rats were coated with a solvent (ethanol: propylene glycol = 3:7) on their bare back skin. Group 3 (L-Lu): The bare back skin of rats was coated with 60 mg/kg/day luteolin (solvent: ethanol: propylene glycol = 3:7). Group 4 (H-Lu): The bare back skin of rats was coated with 120 mg/kg/day luteolin (solvent: ethanol: propylene glycol = 3:7). Group 5 (VE): The bare back skin of rats was coated with 500 mg/kg/d Vitamin E, and the solvent used to dissolve luteolin and Vitamin E was ethanol: propylene glycol = 3: 7. After 30 min of application, rats were exposed to UVB irradiation for 1 h every other day for 1 month.

The UVB radiation source was a device with four UVB lamps (Huangqiang, Nanjing, China). A distance of 20 cm was kept between the irradiated skin of the rats and the lamp source in order to maintain the required radiation dose (300 mJ/cm^2^) for 1 h (Che et al., 2017). At the end of the experiment, the rats were executed and the blood from the abdominal aorta was centrifuged to extract the serum, while the skin tissue from the irradiated area was preserved and stored at −20°C until use.

### Histopathological Analysis

Skin tissue was fixed in 4% paraformaldehyde, then dehydrated, embedded and cut into samples of approximately 4 μm thickness. After removal of paraffin, hematoxylin-eosin (HE) staining and Masson’s staining were performed separately. Histopathological changes in individual samples were observed by light microscopy.

### Immunofluorescence

Skin samples were fixed in 4% paraformaldehyde for 20 min and permeabilised with 0.5% Triton X-100 for 20 min, then blocked with QuickBlock™ Immunostaining Blocking Solution (P0260, Beyotime) for 15 min. The prepared samples were incubated overnight with anti-MMP-1 and anti-Collagen I antibodies at 4°C, then co-incubated with goat anti-rabbit IgG antibody at 37°C for 1 h, followed by continued incubation with DAPI for 5 min at room temperature in darkness. Finally, a fluorescence microscope image system was used for photographic detection of MMP-1 and Collagen I in skin tissue.

### The Detection of Reactive Oxygen Species for Skin Tissue and Cell

A red fluorescent reactive oxygen probe, DHE, can be used to detect the ROS level in skin tissues. Briefly, 10 μlof the DHE probe was added to 190 μl of skin tissue homogenate supernatant, mixed and incubated at 37°C for 30 min in darkness. The samples were then immediately photographed under a fluorescent microscope.

DCFH-DA staining can be used to detect the level of intracellular ROS present in the cell. Briefly, 10 μM DCFH-DA was added to treated cells and incubated in the dark for 1 h. Cells were then washed with pre-cooled PBS and DCFH-DA positivity was measured by flow cytometry.

### Apoptosis Staining

Mitochondrial membrane alterations are thought to be the initial event in apoptosis. JC-1 is a lipophilic cationic fluorescent dye. In normal mitochondria, JC-1 forms polymers that emit intense red fluorescence; in unhealthy mitochondria, due to a decrease or loss of membrane potential, JC-1 can only be present as a monomer, producing green fluorescence. The degree of mitochondrial depolarisation was measured by the ratio of red/green fluorescence intensity. Samples were treated with JC-1 according to the manufacturer’s instructions and the images were then captured by fluorescence microscopy.

### Detection of Malondialdehyde, Superoxide Dismutase, Lactate Dehydrogenase and Senescence-Associated Beta-Galactosidase

MDA, SOD and LDH levels were determined using assay kits. The positive rate of senescent cells can be detected by staining with the SA-β-Gal kit. The assay procedure was carried out in strict accordance with the kit instructions.

### Cells and Modeling

Human dermal fibroblasts (HDF) cells were obtained from Nanjing University of Chinese Medicine. HDF cells were incubated in DMEM medium containing 1% antibiotics (penicillin and streptomycin), 10% fetal bovine serum (FBS) at 37°C in 5% CO_2_. After the cells had reached maximum fusion, they were passaged and used for subsequent *in vitro* experiments.

UVB readily penetrates the epidermis and is almost completely absorbed by the dermis. Therefore, HDF cells are often used to build cellular models to assess the effects of UVB on the skin. HDF cells were irradiated with UVB at a dose of 20 mJ/cm^2^ to construct an *in vitro* acute photoaging cell model (Lee et al., 2018). *In vitro* experiment was divided into five groups: control group, model group (UVB irradiation), 10 μM luteolin group (UVB irradiation with 10 μM luteolin treatment), 20 μM luteolin group (UVB irradiation with 20 μM luteolin treatment), 40 μM luteolin group (UVB irradiation with 40 μM luteolin treatment).

### Cell Viability Assay

HDF cells were placed in 96-well plates (5,000 cells/well) and incubated for 24 h. HDF cells were then treated with three concentrations (10, 20, 40 μM) of luteolin for 2 h and then irradiated with UVB radiation at 20 mJ/cm^2^. After treatment, the cells were incubated at 37°C for 24 h at 5% CO_2_. Cell viability was assessed by MTT analysis. Briefly, MTT at a concentration of 100 μg/ml was added to all wells and then incubated for a further 4 h, and 200 μl DMSO was then added to all wells and mixed thoroughly. Optical density was measured by enzyme calibrator at 490 nm.

### Western Blot Analysis

Tissues and cells were processed to obtain proteins for 10% SDS-PAGE and proteins were transferred onto pre-activated polyvinylidene difluoride (PVDF) membranes. Protein-containing membranes were sealed with 5% skimmed milk powder and incubated overnight at 4°C with specific primary antibodies. The membranes were washed with TBST and incubated with the corresponding horseradish peroxidase-coupled secondary antibody at 37°C for 1 h. ECL chemiluminescent solution was used for color development and photographed using a Bio-Rad gel imaging system. The housekeeping protein Tubulin was used as a reference in this study.

### Statistical Analysis

All measurements and data were performed in three separate experiments. Results were analysed by GraphPad Prism 8.0 software (ANOVA) and are shown as mean ± standard deviation (Mean ± SD). A comparison between the two groups was performed by independent Student’s t-test and ANOVA was used to compare groups. A value of *p* < 0.05 indicates a significant difference.

## Results

### Luteolin Protects Rat Skin From Ultraviolet Radiation B Damage

**FIGURE 1:**
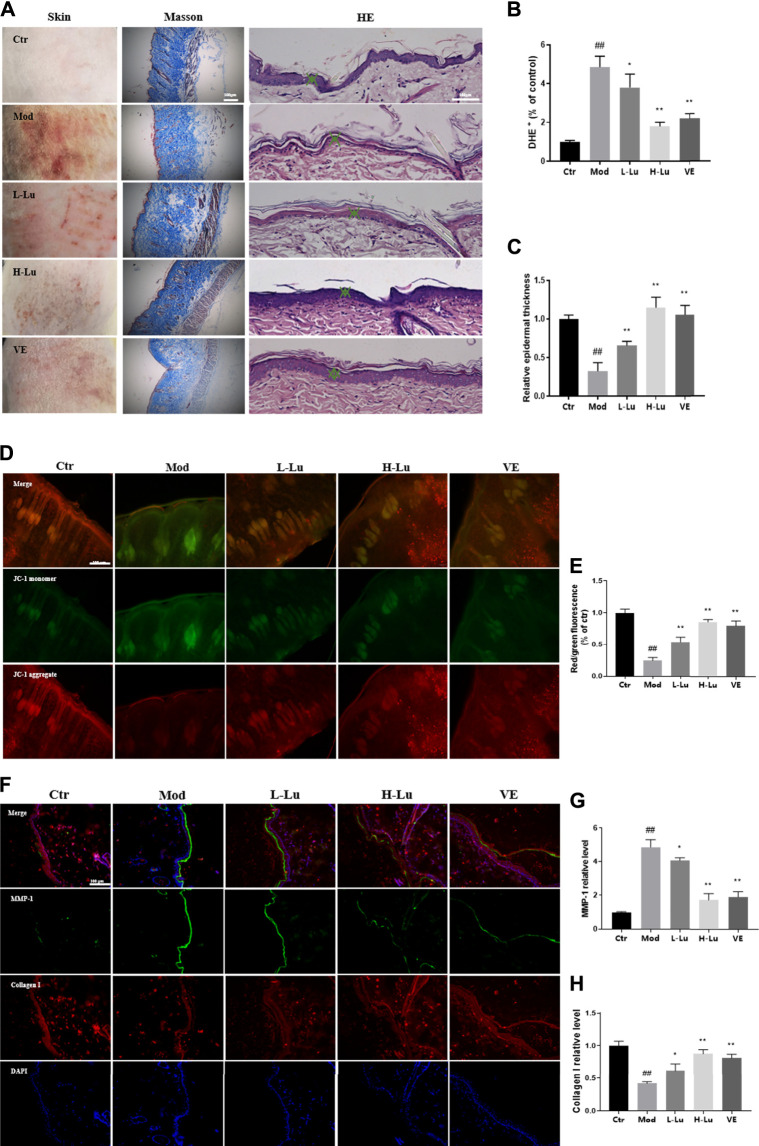
Luteolin improves the condition of the skin on the back of rats damaged by UVB irradiation. **(A)** Changes in skin appearance, Masson and HE sections of rats in each experimental group. **(B)** The content of ROS in the skin of rats in each experimental group, red fluorescence indicated ROS levels. **(C)** The relative epidermal thickness of skin in each group. **(D)** JC-1 staining results of skin in each group, green represents monomers and red represents aggregate. **(E)** The red/green fluorescence ratio of skin in each group, the lower the ratio the lower the membrane mitochondrial potential. **(F)** Representative image of immunofluorescence staining for MMP-1 and Collagen I. **(G)** The relative fluorescent intensity of MMP-1 of skin in each group. **(H)** The relative fluorescent intensity of Collagen I of skin in each group. # indicates significant difference between the model group and the blank group (#*p* < 0.05; ##*p* < 0.01); * indicates significant difference between the administered group and the model group (*p < 0.05; **p < 0.01).

We observed the changes in the bare back skin of rats under UVB irradiation. In Figure 1A, UVB radiation caused erythema and wrinkle damage to the skin. The skin tissue of the model rats showed severe collagen loss, fragmentation and breakage of collagen fibers, and hair follicles in a degenerative and resting state. H&E staining showed that the epidermis of the model group was significantly thinner and the cells were not well arranged (Figures 1A,C). As shown in Figure 1B, DHE fluorescence was significantly enhanced in the model group compared to the control group, implying a higher level of ROS in the skin tissue of the model group compared to the control group. However, topical treatment with luteolin significantly reduced the accumulation of ROS and damage to the skin caused by UVB irradiation. In UVB-irradiated tissues, the mitochondrial membrane potential changed from the aggregate form (red fluorescence) to the monomer form (green fluorescence), as detected using JC-1 staining (Figure 1D). However, luteolin increased the red/green fluorescence ratio, representing a suppression of the UVB-induced disruption of mitochondrial membrane potential (Figure 1E). Further immunofluorescence analysis of the tissue sections showed substantial loss of collagen and increased expression of matrix metalloproteinase-1 (MMP-1) (Figure 1F). There was a significant difference between the luteolin treatment and model groups, with the loss of collagen being alleviated (Figures 1G,H).

### Luteolin Inhibits Ultraviolet Radiation B-Induced Oxidative Damage to the Skin

**FIGURE 2:**
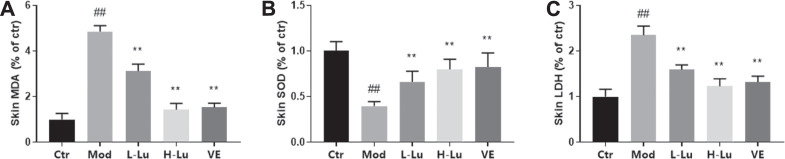
Luteolin protects the skin from UVB-induced oxidative damage. **(A)** The contents of MDA in skin tissues. **(B)** The contents of SOD in skin tissues. **(C)** The contents of LDH in skin tissues. # indicates significant difference between the model group and the blank group (#*p* < 0.05; ##*p* < 0.01); * indicates significant difference between the administered group and the model group (*p < 0.05; **p < 0.01).

Oxygen free radicals act on lipids to produce lipid peroxidation, including MDA, as shown as Figure 2A, where UVB radiation induced oxidative stress in rat skin with a significant increase in MDA levels. SOD is a superoxide anion scavenger enzyme with an important role in biological antioxidant systems. SOD was significantly reduced in rats’ skin after UVB irradiation, indicating that UVB increased the level of oxidative stress within the skin tissue (Figure 2B). The disruption of cell membrane structure due to apoptosis or necrosis leads to the release of enzymes from the cell plasma into the culture medium, including the stable enzymatic activity of LDH. In Figure 2C, results indicated that UVB also induced an increase in LDH as measured in tissue homogenates. However, coating with luteolin significantly restored SOD levels, while significantly reducing MDA and LDH levels in skin tissues.

### Luteolin Inhibits Ultraviolet Radiation B-Induced Activation of Matrix Metalloproteinases and p38 *in vivo*

**FIGURE 3:**
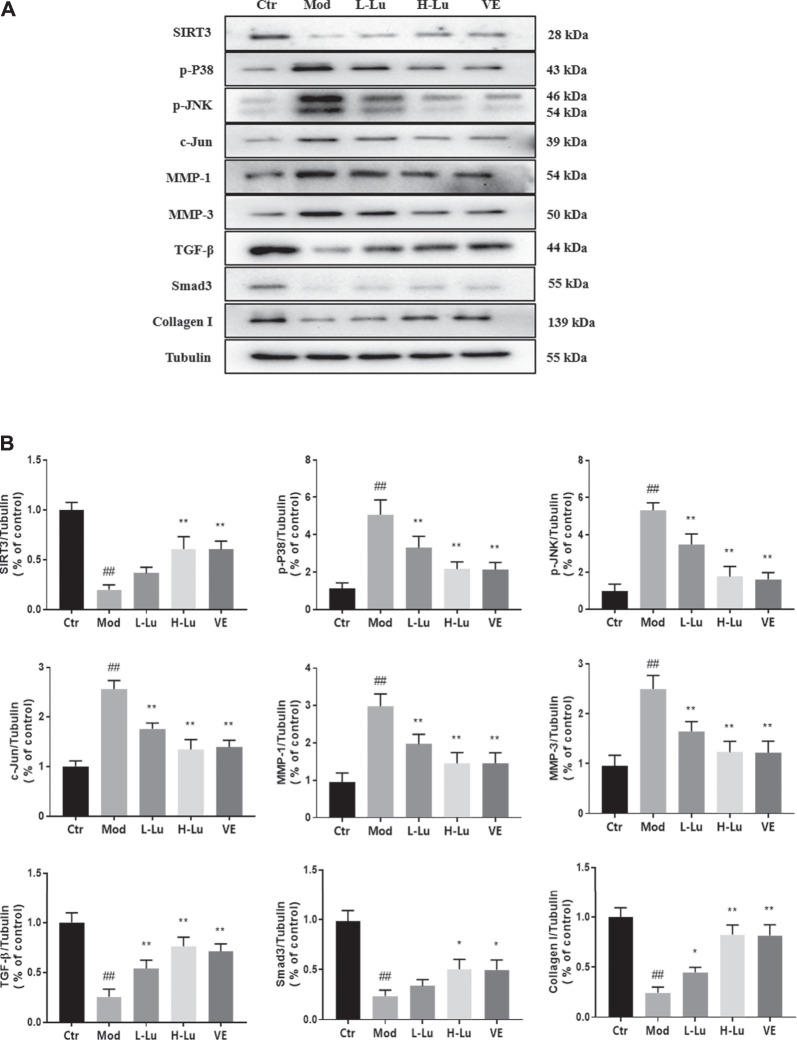
Luteolin inhibits UVB-induced activation of MMPs and p38 MAPK *in vivo*. **(A)** Representative immunoblots of SIRT3, p-P38, p-JNK, c-Jun, MMP-1, MMP-3, TGF-β, Smad3 and Collagen I protein expressions in skin tissue. **(B)** Optical density histograms of SIRT3/Tubulin, p-P38/Tubulin, p-JNK/Tubulin, c-Jun/Tubulin, MMP-1/Tubulin, MMP-1/Tubulin, MMP-3/Tubulin, TGF-β/Tubulin, Smad3/Tubulin and Collagen I/Tubulin. # indicates significant difference between the model group and the blank group (#*p* < 0.05; ##*p* < 0.01); * indicates significant difference between the administered group and the model group (*p < 0.05; **p < 0.01).

Expression of p38 and MMPs in rat skin was detected by Western Blot after UVB irradiation. UVB irradiation resulted in significant increases in p-P38, p-JNK, c-Jun, MMP-1 and MMP-3 and significant decreases in SIRT3, TGF-β, Smad3 and Collagen I (*p* <0.05, Figures 3A, B). These two trends were reversed after luteolin administration, suggesting that luteolin could inhibit the activation of the p-P38 /p-JNK pathway *in vivo* and reduce the production of MMPs, thereby reducing UVB -induced collagen reduction.

### Luteolin Inhibits Ultraviolet Radiation B-Induced Oxidative Damage to the Cell

**FIGURE 4:**
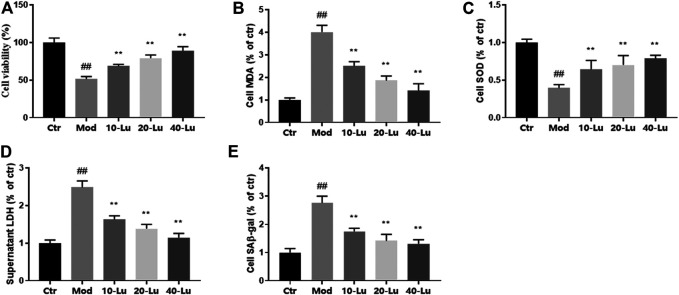
Luteolin improves biochemical parameters on HSF cells exposed to UV radiation. **(A)** Cell viability at different concentrations of luteolin. **(B–D)**, The contents of MDA, SOD and LDH in skin tissues. **(E)** luteolin decreased the content of SAβ-gal of cells under UVB irradiation. # indicates significant difference between the model group and the blank group (#*p* < 0.05; ##*p* < 0.01); * indicates significant difference between the administered group and the model group (*p < 0.05; **p < 0.01).

Cell viability assay was used to detect whether luteolin inhibited UVB-induced cell death, and results showed that luteolin restored the decreased viability of cells caused by UVB-irradiation compared to untreated UVB-irradiated cells (Figure 4A). As shown in Figures 4B–D, the trends of MDA, SOD, and LDH *in vitro* were the same as those *in vivo*, indicating that luteolin could decrease oxidative stress level *in vitro*. The level of SA-β-Gal can be used to indicate cellular senescence, as shown in Figure 4E, where SA-β-Gal level was higher in the model group compared to that in the control group, and the SA-β-Gal level after luteolin treatment.

### Luteolin Increases Intracellular Collagen I Expression and Reduces Reactive Oxygen Species Accumulation

**FIGURE 5:**
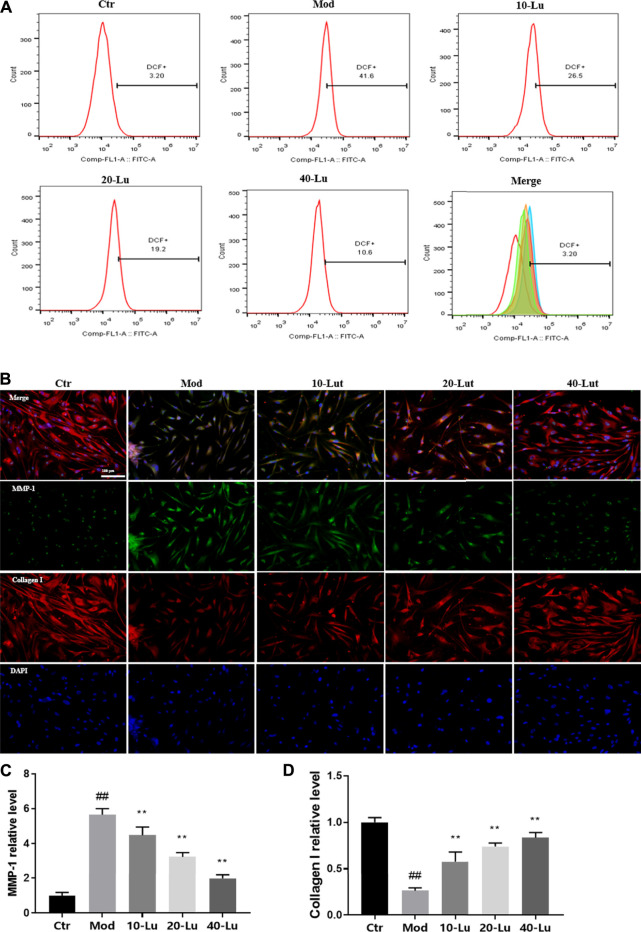
Luteolin increases intracellular Collagen I expression and reduces ROS accumulation. **(A)** luteolin reduced intracellular ROS accumulation. **(B)** Representative image of immunofluorescence staining for MMP-1 and Collagen I. **(C)** luteolin reduced intracellular levels of MMP-1. **(D)** luteolin increased intracellular levels of Collagen I. # indicates significant difference between the model group and the blank group (#*p* < 0.05; ##*p* < 0.01); * indicates significant difference between the administered group and the model group (*p < 0.05; **p < 0.01).

In Figure 5A, luteolin was also found to decrease intracellular ROS levels by flow detection of the DCF probe, which is consistent with the meaning of the trend expressed in Figures 4B,C. As shown by Figures 5B–D, luteolin could dose-dependently decrease the relative immunofluorescence intensity of MMP-1 and increase the immunofluorescence intensity of Collagen I of cells compared to the model group.

### Luteolin Increases Intracellular Collagen I Expression and Reduces Reactive Oxygen Species Accumulation

**FIGURE 6:**
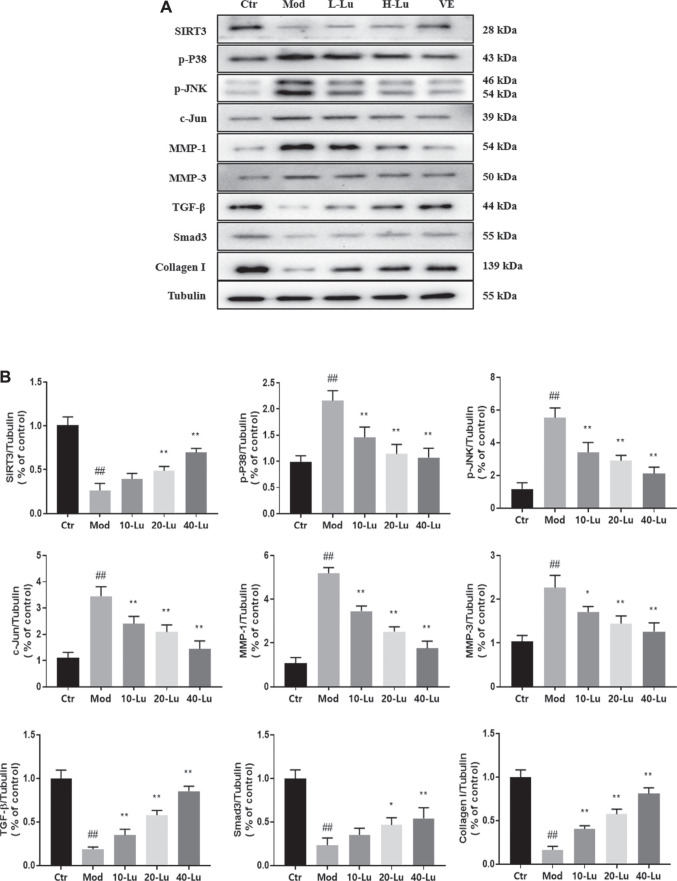
Luteolin inhibits UVB-induced activation of MMPs and p38 MAPK *in vitro*. **(A)** Representative immunoblots of SIRT3, p-P38 MAPK, p-JNK, c-Jun, MMP-1, MMP-3, TGF-β, Smad3 and Collagen I protein expressions in cell. **(B)** Optical density histograms of SIRT3/Tubulin, p-P38/Tubulin, p-JNK/Tubulin, c-Jun/Tubulin, MMP-1/Tubulin, MMP-3/Tubulin, TGF-β/Tubulin, Smad3/Tubulin and Collagen I/Tubulin. # indicates significant difference between the model group and the blank group (#*p* < 0.05; ##*p* < 0.01); * indicates significant difference between the administered group and the model group (*p < 0.05; **p < 0.01).

The expressions of p38 and MMPs in cells after UVB irradiation was also examined in cells by Western Blot. Consistent with the trend in animal experiments, the expressions of p-P38, p-JNK, c-Jun, MMP-1 and MMP-3 were significantly increased and the expressions of SIRT3, TGF-β, Smad3 and Collagen I were significantly decreased after UVB irradiation (Figure 6A). When cells were exposed to UVB irradiation, the luteolin treatment group was involved in the p38/JNK pathway through the upregulation of p-P38 and p-JNK, which in turn inhibited the expression of MMPs and promoted the expressions of Smad3 and collagen I compared to the control group (*p* <0.05, Figure 6B).

### Luteolin Targets and Promotes Sirtuin 3 to Protect Cells From Ultraviolet Radiation B-Induced Damage

**FIGURE 7:**
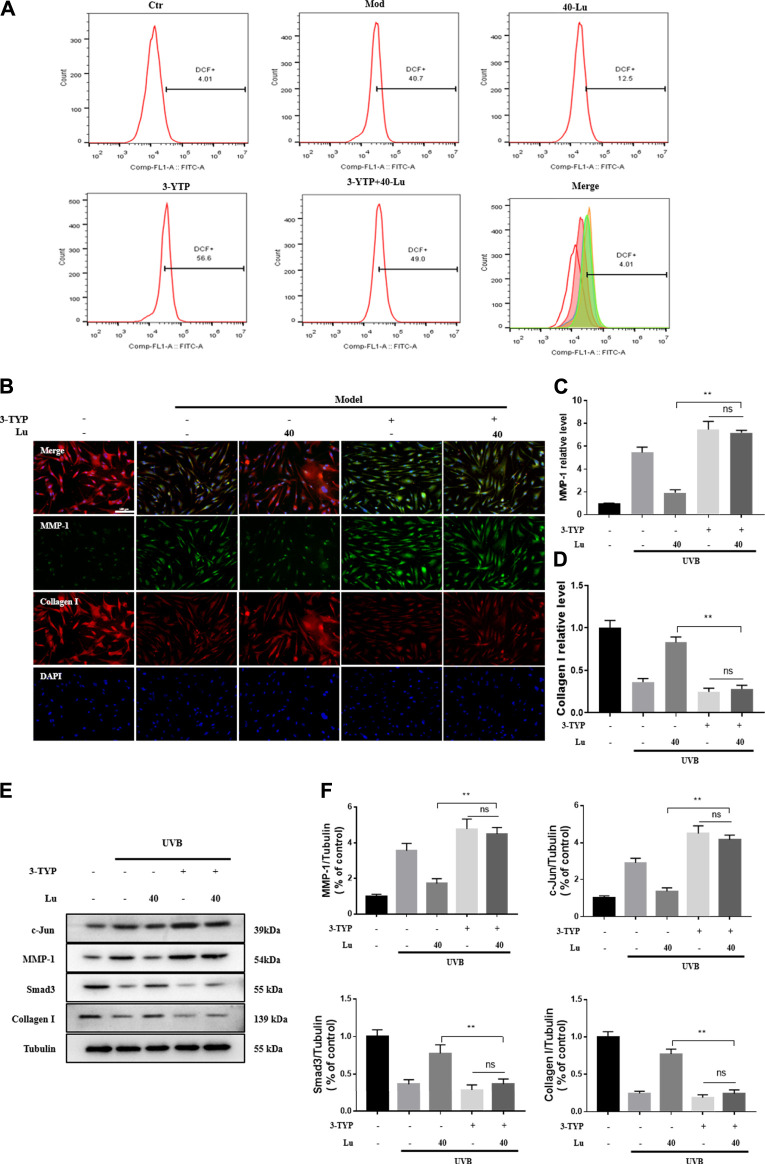
3-TPY counteracts Luteolin’s protection of cells from UVB-induced damage. **(A)** 3-TPY inhibited the inhibitory effect of luteolin on intracellular ROS accumulation. **(B)** Representative image of immunofluorescence staining for MMP-1 and Collagen I. **(C)** 3-TPY inhibited the inhibitory effect of luteolin on intracellular MMP-1 expression. **(D)** 3-TPY inhibited the promotion of luteolin on intracellular Collagen I expression. **(E)** Representative immunoblots of c-Jun, MMP-1, Smad3 and Collagen I protein expressions in cell. **(F)** Optical density histograms of c-Jun/Tubulin, MMP-1/Tubulin, MMP-1/Tubulin, Smad3/Tubulin and Collagen I/Tubulin. * indicates a significant difference between the two groups (*p < 0.05; **p < 0.01).

As seen in Figure 7A, the reduction of intracellular ROS by luteolin was counteracted by the addition of 3-TYP (an inhibitor of SIRT3)*.* Likewise, the inhibitory or promotional effects of luteolin on the protein was counteracted (Figures 7B–F). The above results indicated that the regulation of p38MAPK/JNK signaling by luteolin was inhibited after 3-TYP inhibited the expression of SIRT3.

## Discussion

In this study, we examined skin and cells exposed to UVB irradiation using various indicators related to cellular damage and oxidative stress. For the first time, we demonstrated significant protective effects of luteolin against UVB-induced cell and skin damage in terms of cell viability, oxidative stress, collagen production and erythematous wrinkles. Furthermore, luteolin appears to purposefully promote the regulation of the ROS/p38MAPK axis by SIRT3, which in turn affects the expression of proteins related to the promotion of collagen synthesis and catabolism.

UV radiation is known to negatively affect enzymatic and non-enzymatic antioxidant defences involved in the formation of ROS. It has been shown that topical application of antioxidants protects the skin from oxidative damage (Oresajo et al., 2012) and therefore antioxidants may be considered as an effective means of countering the effects of UV on the skin. UVB-exposed skin treated with and without luteolin was examined and the changes observed are shown in Figure 1A, where UVB exposure had a significant damaging effect on the epidermal layer in the skin, while skin treated with luteolin showed significant improvement. In addition, luteolin had a free radical scavenging effect, reducing the accumulation of ROS in tissues and cells, inhibiting the activity of substances such as LDH and MDA in cells, and enhancing the enzymatic activity of antioxidant enzymes such as SOD, in line with the results of previous studies (Pietta, 2000; Sadik et al., 2003; Choi et al., 2008).

In early UVB irradiation exposure, ROS production leads to the rapid activation of mitogen-activated protein kinases (MAPKs), which are mainly divided into as extracellular signal-regulated kinases (ERKs), C-Jun N-terminal kinases (JNKs) and p38 kinases (Drigotas et al., 2013). The activated protein 1 (AP-1) transcription factor consists of c-Fos and c-Jun. JNKs and ERKs activate phosphorylation of the c-Jun and AP-1 promoters, while p38 kinase plays a key role in the phosphorylation of AP-1 and c-Fos transcription factor subunits (Tanos et al., 2005). As the downstream effector of MAPK, AP-1 can increase the activation of MMPs to reduce collagenase expression (Hwang et al., 2018). Elevated ROS levels can also inhibit the transforming growth factor-β (TGF-β)-promoted collagen production pathway by reducing transforming growth factor-β receptor (TβRII) and SMAD3 protein levels (He et al., 2014).

Skin photoaging is characterized by skin erythema and dryness and deepening of wrinkles. Whereas wrinkles are associated with excessive collagen degradation and fragmentation (Ganceviciene et al., 2012). The decrease in collagen is mediated by two main mechanisms, the induction of collagen degradation due to MMP upregulation (Khalil, 2018) and the decrease in collagen synthesis due to downregulation of TGF-β1, a stimulator of Collagen I (Quan et al., 2004). MMPs are responsible for the degradation and remodeling of each component of the connective tissue extracellular matrix (ECM) (Xiao et al., 2020). Our results show that luteolin reduces the expression of UVB-stimulated MMPs and correspondingly, the expression of collagen is increased, suggesting a potential application of luteolin in wrinkle reduction.

Sirtuin 3 (SIRT3) belongs to the large family of NAD^+^-dependent lysine deacetylases (KDAC) and is the major KDAC in mitochondria, the primary organelle for ROS production (Finkel et al., 2000; Naia et al., 2021). SIRT3 deacetylates some mitochondrial proteins, increases ROS scavenging enzyme activity, inhibits ROS accumulation in mitochondria and stabilizes mitochondrial function (Zhou et al., 2014; Yang et al., 2015). We measured the membrane potential of mitochondria in the skin of each group of rats using the JC-1 probe, as shown in Figures 1D,E, the red/green fluorescence intensity increased in the luteolin-administered group, indicating that luteolin could stabilize mitochondrial function. We further used DHE and DCF fluorescent probes to confirm that luteolin reduced ROS in tissues and cells (Figure 1B and Figure 5A). In addition, it was shown that the reduction of ROS by luteolin was counteracted when 3-TYP, an inhibitor of SIRT3, was combined with luteolin. These results suggest that luteolin stabilises mitochondrial function by targeting and promoting SIRT3 expression to reduce ROS accumulation.

In conclusion, it is reasonable to conclude that luteolin protects skin cells from oxidative stress by reducing the downregulation of SIRT3 expression. After oxidative stress was reduced, the MAPK/MMPs pathway was inhibited, the TGF-β/Smad3 pathway was promoted, and consequently collagen expression was increased.

## Abbreviations

AP-1, activated protein 1; ECM, extracellular matrix; ERKs, extracellular signal-regulated kinases; FBS, fetal bovine serum; HE, hematoxylin-eosin; HDF, human dermal fibroblasts; HRP, horseradish peroxidase; LDH, lactate dehydrogenase; MAPK, mitogen-activated protein kinases; MDA, Malondialdehyde; MMP, matrix metalloproteinase; PVDF, polyvinylidene difluoride; ROS, reactive oxygen species; SA-β-Gal, senescence-associated beta-galactosidase; SIRT3, Sirtuin 3; SOD, superoxide dismutase; TGF-β, transforming growth factor-β; TβRII, transforming growth factor-β receptor; UVB, ultraviolet radiation B; JNKs, C-Jun N-terminal kinases

## Data Availability

The original contributions presented in the study are included in the article/Supplementary Material, further inquiries can be directed to the corresponding author.
