# Supplementary figures and images for: Luteolin Prevents UVB-Induced Skin Photoaging Damage by Modulating SIRT3/ROS/MAPK Signaling: An in vitro and in vivo Studies
Source: Front Pharmacol. 2021 Aug 30;12:728261. doi: 10.3389/fphar.2021.728261 (PMC8436182; doi:10.3389/fphar.2021.728261)

Fig. 3A

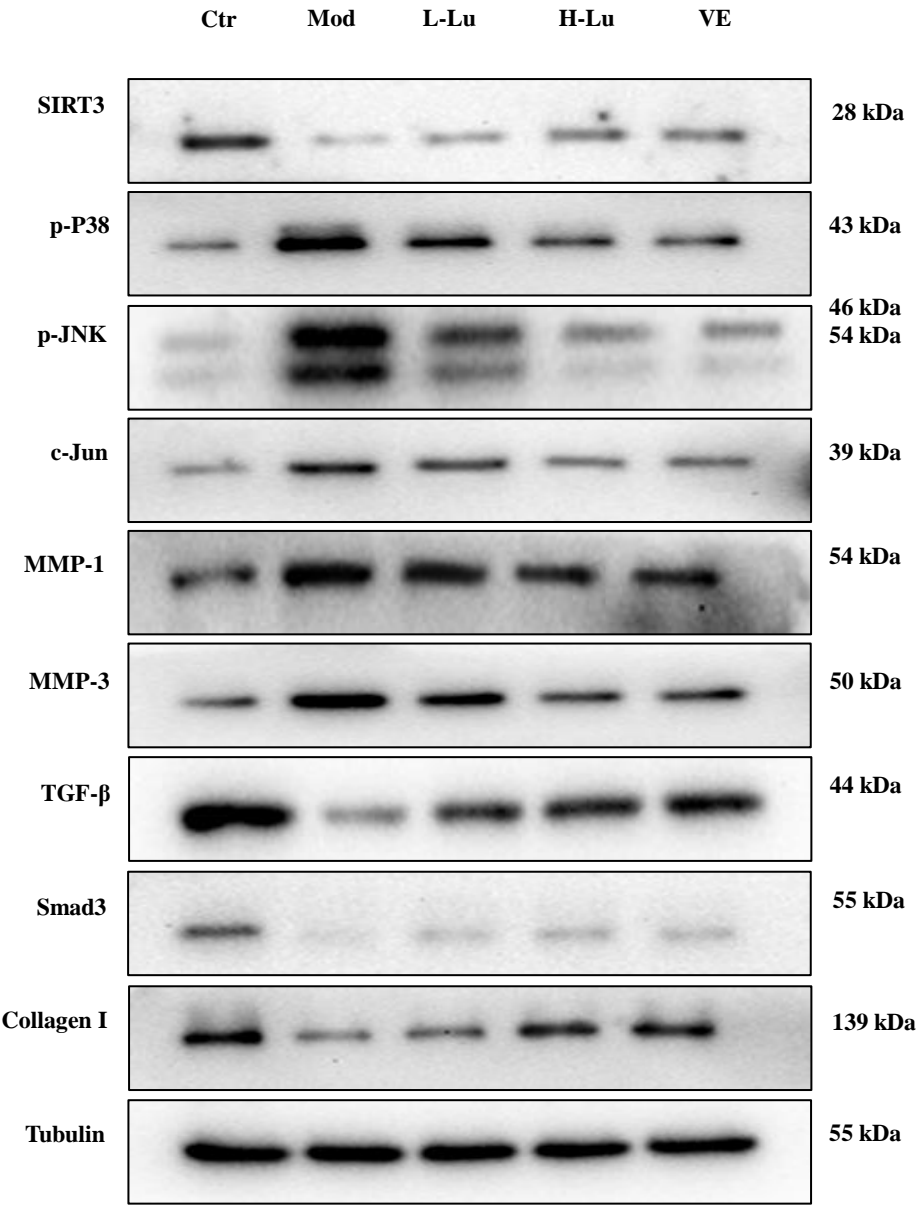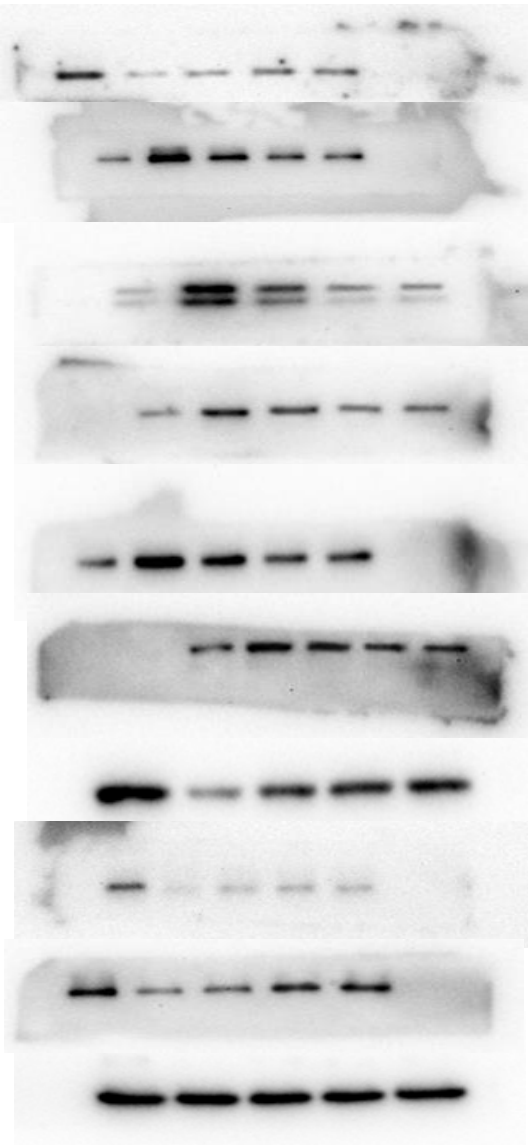

Fig. 6A

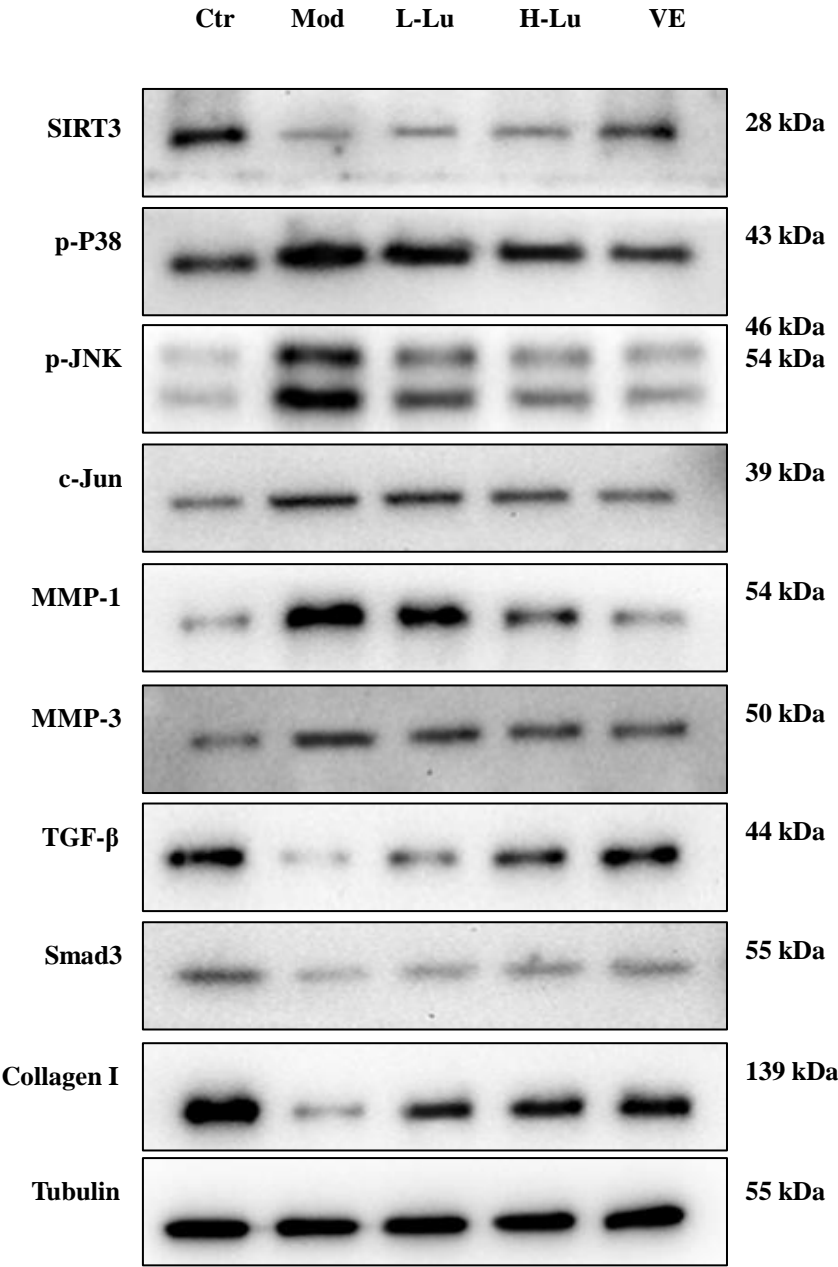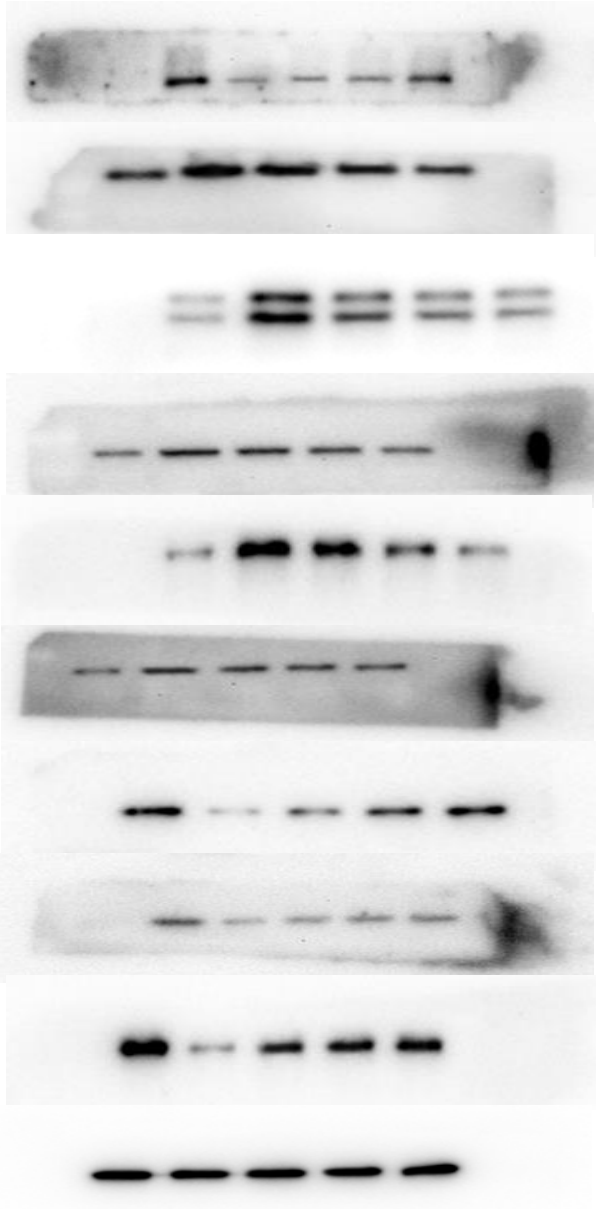

Fig. 7E

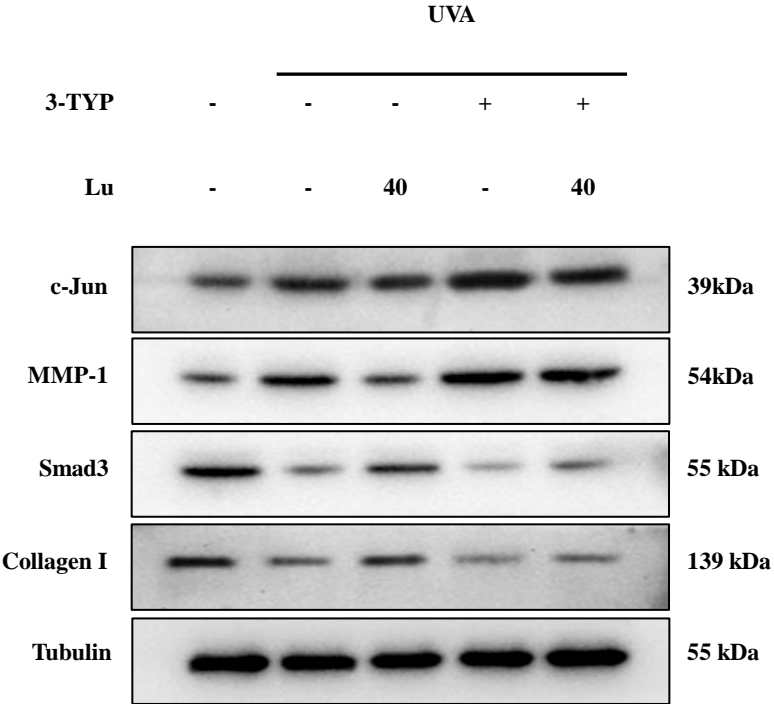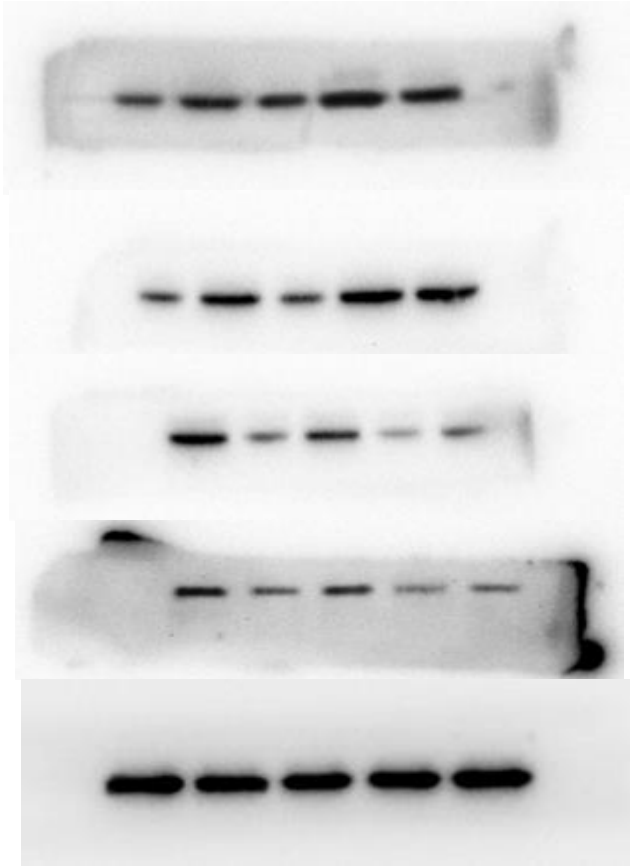

Supplement: Supplementary file 1 [file Image1.PDF]
